# Energy Metabolism in *IDH1* Wild-Type and *IDH1*-Mutated Glioblastoma Stem Cells: A Novel Target for Therapy?

**DOI:** 10.3390/cells10030705

**Published:** 2021-03-22

**Authors:** Cornelis J.F. van Noorden, Vashendriya V.V. Hira, Amber J. van Dijck, Metka Novak, Barbara Breznik, Remco J. Molenaar

**Affiliations:** 1 Department of Genetic Toxicology and Cancer Biology, National Institute of Biology, Večna Pot 111, 1000 Ljubljana, Slovenia; vashendriyavvhira@gmail.com (V.V.V.H.); metka.novak@nib.si (M.N.); barbara.breznik@nib.si (B.B.); r.j.molenaar@amsterdamumc.nl (R.J.M.); 2 Department of Medical Biology, Amsterdam UMC Location Academic Medical Center, University of Amsterdam, 1105 AZ Amsterdam, The Netherlands; a.j.vandijck@amsterdamumc.nl; 3 Department of Medical Oncology, Amsterdam UMC Location Academic Medical Center, University of Amsterdam, 1105 AZ Amsterdam, The Netherlands

**Keywords:** glioblastoma stem cells, *IDH1-*mutation, energy metabolism

## Abstract

Cancer is a redox disease. Low levels of reactive oxygen species (ROS) are beneficial for cells and have anti-cancer effects. ROS are produced in the mitochondria during ATP production by oxidative phosphorylation (OXPHOS). In the present review, we describe ATP production in primary brain tumors, glioblastoma, in relation to ROS production. Differentiated glioblastoma cells mainly use glycolysis for ATP production (aerobic glycolysis) without ROS production, whereas glioblastoma stem cells (GSCs) in hypoxic periarteriolar niches use OXPHOS for ATP and ROS production, which is modest because of the hypoxia and quiescence of GSCs. In a significant proportion of glioblastoma, isocitrate dehydrogenase 1 (*IDH1*) is mutated, causing metabolic rewiring, and all cancer cells use OXPHOS for ATP and ROS production. Systemic therapeutic inhibition of glycolysis is not an option as clinical trials have shown ineffectiveness or unwanted side effects. We argue that systemic therapeutic inhibition of OXPHOS is not an option either because the anti-cancer effects of ROS production in healthy cells is inhibited as well. Therefore, we advocate to remove GSCs out of their hypoxic niches by the inhibition of their binding to niches to enable their differentiation and thus increase their sensitivity to radiotherapy and/or chemotherapy.

## 1. Introduction

James Watson postulated in 2014 that physical activity prevents diseases such as diabetes, dementia, cardiovascular disease, and some types of cancer [1]. Generation of low levels of reactive oxygen species (ROS) during physical activity induce redox potentials that are needed to correctly fold proteins in the endoplasmic reticulum. Therefore, Watson named these diseases redox diseases [1]. We took this hypothesis further with respect to cancer by postulating that this mechanism explains the considerable evidence that physical activity may not only reduce the risk of cancer [2,3], but also prolong the survival of cancer patients, delay the recurrence of cancer [2,4] and improve the quality of life of cancer patients [5].

**Figure 1:**
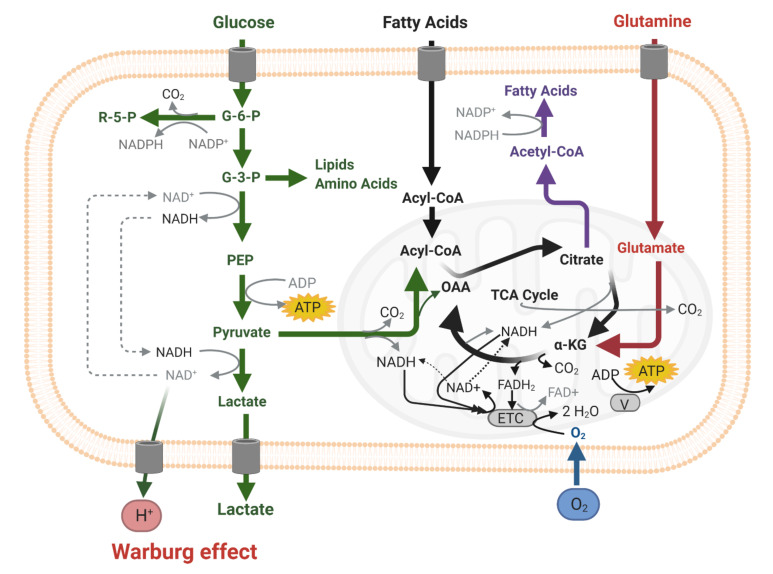
Scheme of cellular energy metabolism (ATP production) in the cytoplasm (glycolysis, green) and in mitochondria oxidative phosphorylation (OXPHOS) via the electron transport chain (ETC, grey) with the use of oxygen (blue). Substrate for glycolysis is (extracellular) glucose and for OXPHOS pyruvate (from the cytoplasm, green), or (extracellular) fatty acids (black), or (extracellular) glutamine and/or glutamate (red).

Proliferating differentiated cancer cells predominantly use glucose in cytoplasmic glycolysis instead of mitochondrial respiration for ATP production, independently of the presence of oxygen (the so-called Warburg effect, Figure 1) [6]. In the past, it was assumed that the Warburg effect occurred because of defective mitochondria in cancer cells, but this is not the case. Proliferating cancer cells preferentially use aerobic glycolysis because besides ATP production, glycolysis enables the synthesis of two elements that are needed by proliferating cells: carbohydrate building blocks and a reduction in the power required for biosynthetic reactions, i.e., NADPH [6,7,8]. ROS shortages may be caused by several mechanisms in proliferating cells. First, the Warburg effect has a negative impact on ROS production, resulting in ROS shortages. ROS are mainly produced during oxidative phosphorylation (OXPHOS) in mitochondria from 0.1–2% of the electrons that escape from the electron transport chain (Figure 1) [9], whereas ATP is mainly generated in the cytoplasmic glycolysis in proliferating cancer cells. Second, NADPH is produced in excess and is not only used for reductive biosynthetic reactions, but also facilitates the antioxidant activity of reduced glutathione [6,7] and detoxifying enzymes [10]. ROS production is elevated during physical activity and that may compensate, resulting in anti-cancer effects. The nuclear factor erythroid 2-related factor 2 (Nrf2) is an important regulator of ROS levels in cells [11]. Nrf2 is activated by ROS and it is degraded by proteasomes after ubiquitinization when ROS levels are low. When Nrf2 is activated, it induces the expression of antioxidant defense systems, including those in the endoplasmic reticulum and in stem cells, such as hematopoietic stem cells in the bone marrow [11].

Besides the rapidly proliferating differentiated cancer cells, tumors also contain a small fraction of undifferentiated cancer stem cells (CSCs) with tumor-initiating and self-renewal properties. CSCs reside in specific hypoxic microenvironments, or niches, where CSCs are maintained in a slowly dividing quiescent state. Quiescence of CSCs protects them from the cytotoxic effects of chemotherapy and radiotherapy, as these therapeutic strategies only target proliferating cells. CSC protection in niches results in tumor recurrence in cancer patients [12,13,14,15,16].

It has become generally accepted that more differentiated (i.e., non-CSC) proliferating cancer cells preferentially use aerobic glycolysis for their ATP production, whereas CSCs preferentially use OXPHOS [12,13,14,15,16,17,18,19,20,21,22]. Similar to healthy cells, CSCs benefit from low levels of ROS, but not excess levels of ROS, which are toxic [23,24]. This is corroborated by the fact that CSCs need hypoxic conditions to control their stem cell fate [15], and the low oxygen levels in the hypoxic niches limit, but certainly do not eliminate, the production of ATP and ROS [14,19]. A similar phenomenon occurs in hematopoietic stem cells in their bone marrow niches [25,26].

On the basis of these facts, a ROS-related riddle becomes apparent. On the one hand, the low levels of ROS that are generated in the context of physical exercise facilitate healthy cells to correctly fold proteins in the endoplasmic reticulum, and thus low ROS levels are beneficial to the human body. On the other hand, it has been postulated that CSCs use mitochondrial respiration for ATP production because low levels of ROS aid CSCs in maintaining their stem cell state [23,24]. How can we solve this riddle from a therapeutical point of view and eradicate CSCs in their protective niches and at the same time allow healthy cells in the bodies of patients to produce low levels of ROS? In the present review, we aim to solve this riddle for glioblastoma patients.

**Figure 2:**
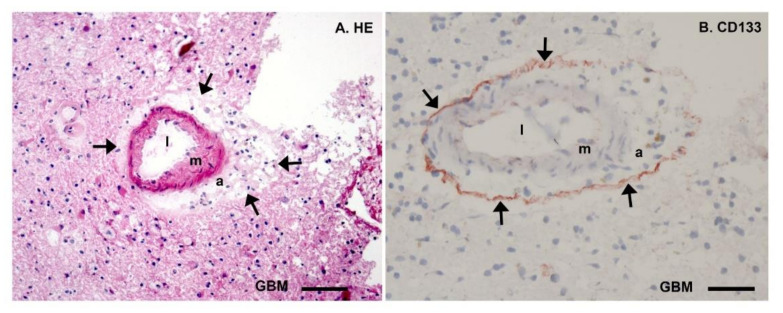
Microscopic images of cryostat sections (8 µm thick) of patient glioblastoma tumor tissue stained with hematoxylin–eosin (HE, (**A**)) and Giemsa combined with immunohistochemical detection of the stem cell biomarker CD133 (arrows, red) showing glioblastoma stem cells (GSCs) in their protective hypoxic peri-arteriolar niche (**B**). Both in (**A**) and (**B**), a cross section of an arteriole is shown with the lumen (l), the tunica media containing smooth muscle (m), the tunica adventitia containing stroma (a) and surrounded by a thin layer of GSCs adjacent to the tunica adventitia in (**B**) and then differentiated glioblastoma cells (GBM). Bars: (**A**), 100 µm and (**B**), 50 µm. Reprinted with permission [24].

In glioblastoma brain tumors, glioblastoma stem cells (GSCs) are protected in their hypoxic peri-arteriolar GSC niches (Figure 2) in a similar way to how healthy HSCs and leukemic stem cells (LSCs) are protected in hypoxic peri-arteriolar niches in the bone marrow [25,26,27,28]. In addition, there are similarities between GSCs in hypoxic peri-arteriolar GSC niches and neural stem cells (NSCs) in the hypoxic subventricular zone (SVZ) and subgranular zone (SGZ) [16,29,30]. GSCs are non-dividing or slowly dividing and, in that quiescent state, have a modest metabolism. GSCs are held responsible for the recurrence of glioblastoma after treatment (surgery, radiotherapy, and temozolomide chemotherapy). Survival of glioblastoma patients is on average 15 months, but recently it was reported that survival of the fittest patient population can be prolonged to 20 months by the application of magnetic tumor-treating fields (TTF) [31]. The canonical molecular anti-cancer mechanism of TTF is that this novel modality disrupts mitosis by interference with heterotrimer septin complexes and α/β-tubulin at the metaphase–anaphase transition in the cell cycle causing mitotic catastrophe [32,33]. In addition, interference with energy metabolism seems plausible because the production of pyruvate, the end-product of glycolysis and a critical fuel for mitochondrial respiration, is reduced when TTF are applied [34].

In order to solve the ROS-related riddle for glioblastoma patients, we compare the energy metabolism in differentiated glioblastoma cells versus that in GSCs, in the presence or absence of the canonical heterozygous isocitrate dehydrogenase 1 mutation (*IDH1*mt). First, we review the present state of affairs with respect to energy metabolism in differentiated glioblastoma cells that are *IDH1* wild-type (*IDH1*wt) or *IDH1*mt. Second, we discuss possibilities to differentially target therapeutically the energy metabolism of *IDH1*wt and *IDH1*mt GSCs in primary and secondary glioblastoma, respectively.

*IDH1* is a metabolic enzyme in the cytoplasm, endoplasmic reticulum, and peroxisomes and the mutation causes a neo-enzymatic activity [10,35,36]. The consequences of this altered activity in differentiated glioblastoma cells are relatively well understood [10], but the consequences of the *IDH1*mt for GSCs are unknown, despite the fact that GSCs are considered to be the prime target for therapy to prevent the recurrence of glioblastoma after therapy [10,16,25,26] and energy metabolism is considered to be an attractive therapeutic target in cancer [37]. Therefore, it is crucial to determine whether quiescent GSCs can be effectively and specifically treated therapeutically with metabolic stressors or inhibitors affecting the energy metabolism in mitochondria. Inhibitors of mitochondrial metabolism for the therapeutic targeting of CSCs have been reviewed recently [12,13,17,18,20,38,39]. It has to be stressed here that the inhibition of aerobic glycolysis, which is a hallmark of cancer [37], in differentiated proliferating glioblastoma cells is not an option for treating glioblastoma patients because clinical trials have thus far shown that potential drugs targeting glycolysis are either not well tolerated or had no clinical efficacy [18,38,40,41].

## 2. Energy Metabolism of *IDH1wt* versus *IDH1mt* Differentiated Glioblastoma Cells

**Figure 3:**
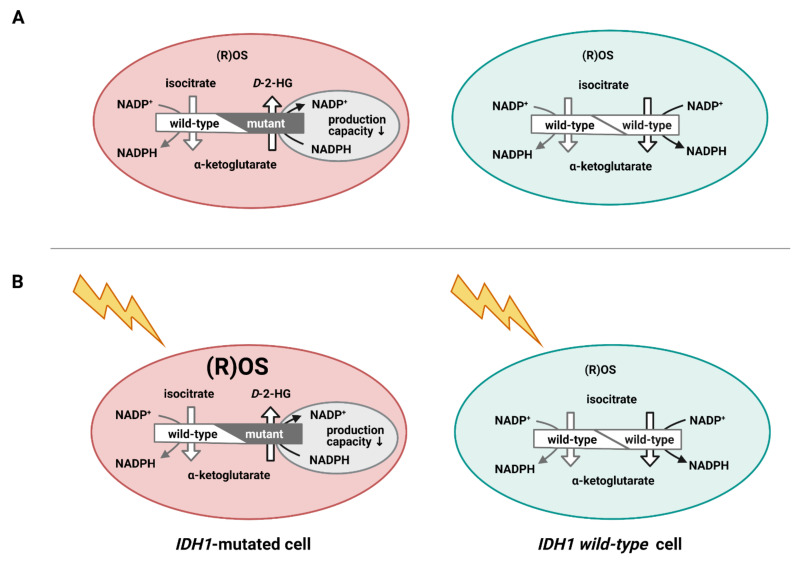
Cartoon of isocitrate dehydrogenase 1 (*IDH1*) wild-type cells (blue) and *IDH1*-mutated cells (pink) showing the functional IDH1 dimer of 2 wild-type alleles and of one wild-type allele and one mutated allele. The wild-type dimer produces α-ketoglutarate and NADPH and the heterozygous mutated dimer produces d-2-hydroxyglutarate (D-2-HG) and NADP^+^. Reactive oxygen species (ROS) levels are in a steady state more or less similar in both cancer cells (**A**), whereas ROS levels accumulate in the *IDH1*-mutated cell due to irradiation because of reduced NADPH production, unlike in the wild-type cancer cell (**B**).

A striking example of metabolic flexibility in gliomagenesis is the metabolic rewiring in *IDH1*mt glioblastoma compared to *IDH1wt* glioblastoma [42]. The *IDH1* mutation occurs at a hot spot of the *IDH1* gene and is the main driver of *IDH1*mt glioblastoma that make up 5% of all glioblastoma tumors [10]. *IDH1*wt converts isocitrate and NADP^+^ into α-ketoglutarate (α-KG) and NADPH, whereas *IDH1*mt converts α-KG and NADPH into the oncometabolite D-2-hydroxyglutarate (D-2-HG) and NADP^+^ (Figure 3). The *IDH1* enzyme functions as a dimer of two *IDH1* proteins and only one of the two proteins is mutated, since a dimer of two mutated proteins is nearly completely inactive and may not confer a survival advantage to the glioma cell [43]. Thus, the *IDH1*wt protein of the dimer produces α-KG and NADPH which are subsequently consumed by the *IDH1*mt protein of the dimer (Figure 3) [10].

Because the capacity to metabolize D-2-HG is low, mainly by D-2-HG dehydrogenase, D-2-HG accumulates in the cells and intracellular concentrations up to 30 mM have been reported [10,44]. The accumulation of D-2-HG and concomitant depletion of α-KG and NADPH cause a plethora of alterations in cells, affecting metabolism, DNA repair, redox state, epigenetics, phospholipid composition, and epigenetics, ultimately leading to gliomagenesis and the development of glioblastoma [10,45]. Moreover, it was reported recently that *IDH1*mt increases the stiffness of the cytoskeleton which reduces the invasive behavior of *IDH1*mt glioblastoma cells [46] which may well be associated with their reduced glycolytic activity (see below). This finding is in line with the mechanical regulation of glycolysis by the cytoskeleton as a response to the composition of the extracellular environment, whereas cancer cells shut down this mechanical regulation and keep glycolytic activity high [47]. A tenascin-c-enriched extracellular matrix in *IDH1*mt glioblastoma enhances its stiffness, thus reducing glioblastoma aggression [48]. However, it should be stressed here that *IDH1*mt glioblastoma cells are extremely difficult to grow in vitro, and thus cells overexpressing *IDH1*mt are used that do not reflect the activity of naturally occurring *IDH1*mt cancer cells, especially because the 1:1 stoichiometry of heterodimers of wild-type and mutated *IDH1* enzymes cannot be reliably replicated with overexpression systems.

For the synthesis of ATP, a thorough metabolic rewiring occurs in *IDH1*mt cells, leading to a vast increase in the number of mitochondria as was shown in oligodendroglioma cells [49]. *IDH1*wt glioblastoma cells mainly use glycolysis as a classical Warburg phenotype that produces lactate [50,51], whereas *IDH1*mt secondary glioblastoma use OXPHOS for the generation of ATP using pyruvate and glutamate, which has been determined at the gene expression, protein, and metabolite levels [42,45,52,53,54,55].

Metabolic rewiring as occurs in *IDH1*mt glioblastoma has also been described as occurring in 3% of glioblastoma patients with fibroblast growth factor receptor 3 (FGFR3)–transforming acidic coiled-coil-containing protein 3 (TACC3) gene fusions [56].

*IDH1*mt is associated with a prolonged survival of glioblastoma patients of approx. 2 years compared to primary glioblastoma [35,36,46]. This is at least partially caused by the increased oxidation of NADPH via *IDH1*mt which renders the affected glioblastoma cells more vulnerable to ROS induced by irradiation and chemotherapy (Figure 3) [46]. NADPH is the major intracellular reducing power to reduce glutathione, thioredoxin, catalase tetramers, and cytochrome P450, all of which are involved in detoxification processes, including those of ROS [7,10] which glioblastoma cells need to survive irradiation and chemotherapy.

**Figure 4:**
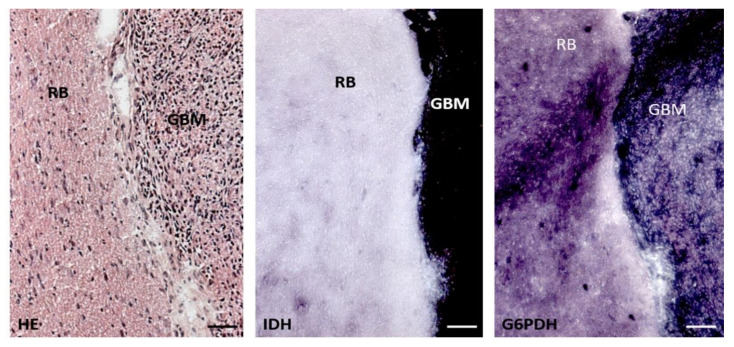
Serial cryostat sections (8 µm thick) of rat brain (RB) containing a patient-derived glioblastoma tumor (GBM) stained with hematoxylin–eosin (HE), and stained with metabolic mapping for isocitrate dehydrogenase (IDH) activity and glucose-6-phosphate dehydrogenase (G6PDH) activity. G6PDH activity is more or less similar in rat brain and human tumor tissue, whereas IDH activity is manifold stronger in human glioblastoma tissue than in rat brain. Bars, 100 µm. Reprinted with permission [50].

In human brain tissue and glioblastoma tumors, IDH1 is the major provider of NADPH [36]. This is not the case in rodents (Figure 4), and *IDH1*mt does not increase the survival of acute myeloid leukemia patients because glucose-6-phosphate dehydrogenase (G6PD) is the major provider of NADPH in white blood cells rather than IDH1 [57,58]. However, prolonged survival of *IDH1*mt glioblastoma patients may not only depend on the insufficient availability of NADPH to detoxify ROS during radiotherapy and chemotherapy because *IDH1*mt-transduced astrocytes and glioma cells have been found to retain stable NADPH levels by replenishing NADP^+^ and NADPH levels via the synthesis of NADP^+^ from NAD^+^ by NAD kinase, thus suggesting additional mechanisms of *IDH1*mt-associated vulnerability to therapy [59]. Gelman et al. reported that in *IDH1*mt-transduced human fetal astrocytes, NADPH production by G6PD is increased for D-2-HG synthesis [60]. However, we did not find elevated G6PD activity in *IDH1*mt versus *IDH1*wt glioblastoma tumor samples of patients [36].

**Figure 5:**
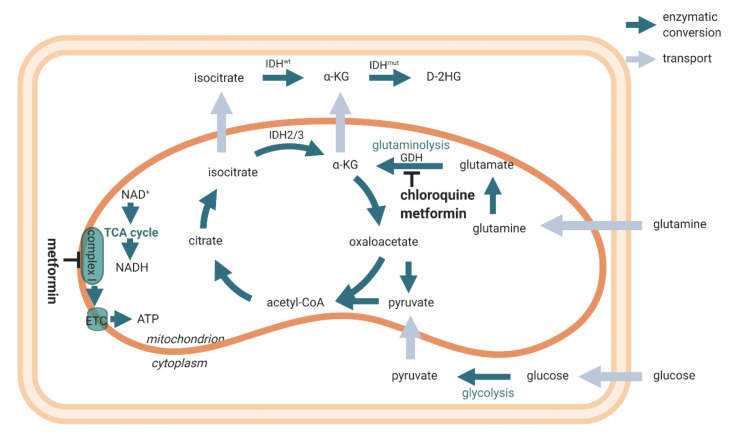
Scheme of cellular energy metabolism (ATP production) and the inhibitory actions of metformin and chloroquine. Reprinted with permission [54].

Phase 1B/II clinical trials are ongoing at present to investigate whether the treatment of *IDH1*mt glioblastoma and other cancer types with the *IDH1*mt gene can be optimized by interfering with the mitochondrial ATP production. For this purpose, patients are treated with the anti-diabetic and FDA-approved drug metformin in combination with the anti-malaria and FDA-approved drug chloroquine [61]. Metformin and its lipophilic analogue phenformin, which may reach higher concentrations in the mitochondria of cancer cells, inhibit complex I of the electron transport chain of OXPHOS, whereas α-KG production from glutamine and glutamate by glutamate dehydrogenase is inhibited by metformin, phenformin, and chloroquine (Figure 5) [10,61].

In conclusion, differentiated *IDH1*wt glioblastoma cells depend on aerobic glycolysis for ATP production, whereas differentiated *IDH1*mt glioblastoma cells import pyruvate and glutamate into mitochondria [42] to fuel OXPHOS for ATP production.

## 3. Energy Metabolism in *IDH1wt* GSCs

The energy metabolism of *IDH1*wt GSCs has not yet been very well studied. However, the general consensus is that GSCs are metabolically flexible but mainly use OXPHOS for the generation of ATP, whereas differentiated *IDH1*wt glioblastoma cells use aerobic glycolysis [51,62,63].

In recent years, a number of proteins has been described that regulate OXPHOS activity in GSCs and may become alternative targets for therapy to shut down OXPHOS in *IDH1*wt GSCs (Table 1). When interpreting these results, it is important to note that Duraj et al. recently reported that GSCs cultured in the absence of serum show heterogeneous energy metabolism and variable responses to inhibitors of cellular metabolism [64].

First, translocator protein (TSPO) is involved in OXPHOS in GSCs. TSPO is a transmembrane protein in the outer mitochondrial membrane and facilitates cholesterol transport across the mitochondrial intermembrane space [65]. In the brain, it is mainly expressed in glial cells [65]. It is also highly expressed in glioma [66]. In human GSCs, the loss of TSPO resulted in a shift from OXPHOS towards glycolysis with an increased glucose uptake and lactate production. Moreover, mitochondria were found to be fragmented after the loss of TSPO, whereas tumor growth intracranially in mice was increased [66]. Therefore, TSPO seems essential for the maintenance of GSCs and thus a promising protein to be targeted therapeutically.

Second, insulin-like growth factor 2 mRNA-binding protein 2 (IGF2BP2 or IMP2) is functional during embryonal development, it is linked with susceptibility to type 2 diabetes and participates in the maintenance of CSCs [67]. Janiszewska et al. [68] reported that OXPHOS is maintained in GSCs by IGF2BP2 that delivers electron transport chain subunit-encoding mRNAs to mitochondria and contributes to complex I and IV assembly. Therefore, IGF2BP2 is another interesting protein to be targeted therapeutically.

Third, glycerol-3-phosphate is a substrate for glycerol synthesis. Glycerol-3-phosphate dehydrogenase I (GPDI) is expressed upon osmotic stress. It is expressed by GSCs but not by NSCs and may well be linked with edema formation in glioblastoma [69]. GSCs express GPDI in relation to their quiescence. An interesting association was made between GSC quiescence and elevated glycerol levels in dormant insects during their development, and in hibernating mammals [69]. It is suggested that GPDI is an attractive therapeutic target to treat glioblastoma as GSC quiescence is inhibited, resulting in increased therapy sensitivity.

Fourth, oncostatin M is a cytokine of the interleukin-6 (IL-6) subfamily and is expressed in the brain by various cell types (neurons, astrocytes, and microglia). It is involved in immunosurveillance in the brain [70]. Its receptor is expressed by GSCs in mitochondria and interacts with complex I to promote OXPHOS. Deletion of the oncostatin M receptor reduces OXPHOS, increases ROS levels, and sensitizes GSCs to irradiation [71]. IL-6 itself induces CD133 expression via the transfer of STAT3 into the nucleus in hypoxic conditions [72].

Various inhibitors of OXPHOS activity of GSCs have been described as well [12,13,17,18,20,63,73] (Table 2).

First, metformin and phenformin are inhibitors of OXPHOS as described above and in Figure 5, and metformin is being tested in clinical trials in patients with cancers with *IDH1mt* [61]. Nuclear magnetic resonance-based metabolomic analysis showed anti-cancer effects of metformin treatment [74].

Second, Mudassar et al. [75] reviewed the role of hypoxia in combination with OXPHOS and resistance of GSCs to irradiation. Their focus was to inhibit OXPHOS to increase the low oxygen levels in hypoxic GSC niches to sensitize GSCs to irradiation. Repurposing of the anti-parasitic drugs atovaquone, ivermectin, proguanil, mefloquine, and quinacrine that inhibit OXPHOS in various ways was their approach to reduce hypoxia in GSC niches and render GSCs more vulnerable to radiotherapy and chemotherapy.

Third, lonidamine (LND) is an anti-glycolytic drug with limited clinical effects in cancer patients [13,38,40,41]. However, LND in a mitochondria-targeting form (Mito-LND) appears to be a selective OXPHOS inhibitor with very low toxicity in mice [41]. These characteristics of Mito-LND makes it an attractive candidate to target CSCs in general and GSCs and *IDH1*mt glioblastoma in particular, as they primarily depend on OXPHOS.

Fourth, verteporfin inhibits OXPHOS at complexes III and IV of the electron transport chain very efficiently (IC_50_ = 200 nM) [76]. It is specifically cytotoxic against GSCs and not to differentiated glioblastoma cells or normal cells. 

Fifth, the effects of a ketogenic diet on patient-derived GSCs were studied in vitro by incubation of GSCs in media containing β-hydroxybutyrate and restricted glucose levels that mimic the clinical effects of such a diet. It was found that ROS levels were increased in GSCs and apoptosis was induced, whereas ROS scavengers annihilated these effects [77]. However, an unrestricted ketogenic diet did not reduce tumor growth in vivo in various glioblastoma mouse models, whereas the inhibition of fatty acid oxidation by etomoxir reduced glioblastoma growth in the same mouse models [50]. It appeared that etomoxir prolonged the survival of mice whereas the ketogenic diet did not affect survival or even reduce the survival of the mice. *IDH1*wt and *IDH1*mt glioblastoma cells were not differently affected by etomoxir [50]. We conclude that the long-standing conviction that a ketogenic diet is beneficial for glioblastoma patients has no scientific grounds. It should not be considered for glioblastoma patients because a ketogenic diet may even have adverse effects on glioblastoma tumor growth. Kant et al. demonstrated that fatty acid oxidation provides β-hydroxybutyrate for ketogenesis that stimulates glioblastoma cell proliferation in vitro and that finding was recapitulated in glioblastoma tumors [51]. Furthermore, a ketogenic diet is hard to maintain and thus has unnecessary negative effects on quality of life of glioblastoma patients.

In conclusion, a number of proteins that are involved in OXPHOS activity have been described recently as potential targets for anti-OXPHOS therapy as well as selective inhibitors of OXPHOS activity in GSCs. These developments certainly deserve follow-up studies to establish whether or not one or more can be developed into an opportunity to therapeutically target *IDH1*wt GSCs in patients.

## 4. Energy Metabolism in *IDH1*mt GSCs

The energy metabolism of GSCs has not been studied in relationship with *IDH1*mt, as far as we know. Therefore, we have to extrapolate from the data that are available regarding energy metabolism in *IDH1*mt and *IDH1*wt glioblastoma tumors in combination with our understanding of the energy metabolism in stem cells in general and in CSCs in particular. In this way, strategies can possibly be formulated for testing in future studies for the rational design of therapies targeting energy metabolism in *IDH1*mt GSCs.

The energy metabolism of *IDH1*mt glioblastoma is forced to be dependent on OXPHOS instead of aerobic glycolysis because of the drain of αKG and NADPH by the *IDH1*mt enzyme that converts αKG into D-2-HG with concomitant oxidation of NADPH into NADP^+^ [42,45,52,53,54,55]. This altered metabolism causes metabolic stress, and it has been hypothesized by us and others that the increased metabolic stress induced by the therapeutic targeting of the mitochondrial energy metabolism prolongs the survival of *IDH1*mt glioblastoma patients [10,50,61]. Candidate compounds in this respect are metformin or phenformin, chloroquine, and epigallocatechin-3-gallate (EGCG) to inhibit the production of αKG by glutamate dehydrogenase. As explained above, metformin and phenformin inhibit complex I of the electron transport chain of OXPHOS and glutamate dehydrogenase, whereas chloroquine inhibits glutamate dehydrogenase (Figure 5) [10,61]. EGCG, a major polyphenol flavonoid in green tea [78], reduces D-2-HG production and the proliferation of *IDH1*mt glioblastoma cells [79]. In glioblastoma patients, EGCG seems effective only when used in large quantities as an adjuvant during radiotherapy and temozolomide chemotherapy [78]. It is worthwhile to investigate whether this adjuvant effect of EGCG is more profound in *IDH1*mt glioblastoma patients than in *IDH1*wt patients.

Glutamate is present extracellularly in high concentrations in brain tissue and can be imported into the mitochondria by glioblastoma cells [80,81]. Moreover, glutamate has been determined as a necessary metabolite for *IDH1*mt glioblastoma [42,45,52,53,54,55,56,57,58,59]. Reduced levels of glutamate in *IDH1*mt-transduced cells [82] confirm the potential of glutamate dehydrogenase inhibition to increase the metabolic stress of *IDH1*mt glioblastoma cells.

**Figure 6:**
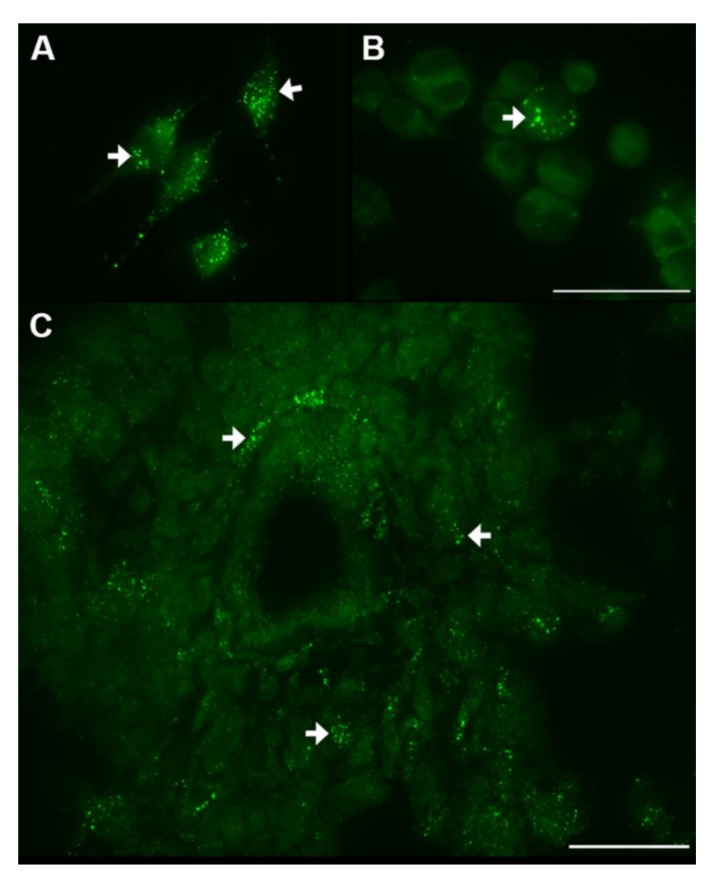
Intracellular activity of cathepsin B in lysosomal-like organelles (strongly green fluorescent dots, white arrows) in cultured differentiated glioblastoma cells (U373) (**A**) and GSCs (**B**) and in GSCs around an arteriole in a cryostat section of a patient-derived glioblastoma tumor (**C**). Localization of cathepsin B activity was performed as described in [83]. Bars, 50 µm.

Inhibition of the conversion of glutamine into glutamate by glutaminase has also been investigated to increase metabolic stress in CSCs. The small-molecular inhibitor CB839 of glutaminase activity eradicates GSCs in neurospheres [40]. Moreover, the glutamine analogue (6-diazo-5-oxo-L-norleucine (DON))-containing prodrug JHU083 inhibits glutamine-metabolizing glutaminase in cancer cells. The prodrug is cleaved by cathepsins that are abundantly present in glioblastoma cells and extracellularly in the microenvironment of tumors (Figure 6) [83,84,85] to release locally the active glutamine analogue DON to inhibit glutamine conversion selectively in cancer cells. This is most relevant because other glutamine-using cells such as T cells are not affected and can still perform their immune anti-cancer function [86].

Moreover, D-2-HG strongly inhibits the transaminases branched-chain aminotransferase 1 (BCAT1) and BCAT2, thus lowering glutamate levels in *IDH1*mt glioblastoma cells. It explains their sensitivity to glutaminase inhibitors because the inhibitors increase metabolic stress in *IDH1*mt cancer cells [87].

It has also been suggested to treat patients with *IDH1*mt glioblastoma with inhibitors of the mutated protein of *IDH1* [88]. However, we want to emphasize that this has to be done with caution because as a consequence of *IDH1*mt inhibition, metabolic stress is reduced and NADPH production capacity is increased, and thus the more effective radiotherapy and chemotherapy in *IDH1*mt glioblastoma patients is lost. Therefore, inhibitors of the *IDH1*mt protein should not be administered to patients during radiotherapy and chemotherapy [89].

In conclusion, experimental data are not yet available of the identity of the energy metabolism in *IDH1*mt GSCs, but it is reasonable to assume that the metabolic differences between *IDH1*mt GSCs and *IDH1*mt differentiated glioblastoma cells are smaller than the metabolic differences between *IDH1*wt GSCs and *IDH1*wt differentiated glioblastoma cells. A major rationale that supports this hypothesis is the finding that the metabolic rewiring in *IDH1*mt differentiated glioblastoma cells is associated with the metabolic rewiring that is also associated with the acquisition of stemness. This assumption should be tested in future studies. If this is the case, there is no need for the specific targeting of the OXPHOS metabolism of *IDH1*mt GSCs because both GSCs and differentiated glioblastoma cells depend on OXPHOS. Furthermore, *IDH1*mt glioblastoma patients should not be treated with inhibitors of the *IDH1*mt protein during radiotherapy or chemotherapy because the inhibitors counteract the vulnerability of *IDH1*mt glioblastoma to therapy.

## 5. Concluding Remarks

Cancer is a redox disease and is closely associated with ATP production in mitochondria (OXPHOS), the source of ROS in cells. In healthy cells in our body, low ROS levels are beneficial and may have an anti-cancer effect because they reduce cancer risk, prolong cancer patient survival, delay cancer recurrence, and improve the quality of life of cancer patients. In glioblastoma, like in many other cancer types, the differentiated glioblastoma cells produce ATP preferentially in cytoplasmic glycolysis, both anaerobic and aerobic. This Warburg effect has been excellently reviewed recently by Vaupel and Multhoff [8]. In this review, the Warburg effect is explained on the basis of current metabolic perspectives as an essential part of a selfish metabolic reprogramming in differentiated cancer cells. Because of the high proliferation rate and sensitivity of differentiated glioblastoma cells to cytotoxic agents, radiotherapy and chemotherapy are effective. Inhibition of glycolysis is not an option in glioblastoma patients because clinical trials of inhibitors of glycolysis have been proven to be either ineffective or causing unwanted side effects.

The small amount of GSCs in glioblastoma tumors and in the SVZ, whether they are *IDH1*wt or *IDH1*mt, preferentially use OXPHOS for ATP production and are well protected against radiotherapy and chemotherapy in their hypoxic niches, mainly because they are slowly proliferating. GSCs have a modest metabolism and produce low levels of ROS. Rapidly increasing numbers of specific therapeutic targets that may cause inhibition of OXPHOS are becoming available in order to attack ATP production in GSCs in particular and in CSCs in general.

However, when the need for OXPHOS in healthy cells in the body of cancer patients is taken into consideration, it must be concluded that systemic treatment of cancer patients with OXPHOS inhibitors is not an option either. It annihilates the benefits of low levels of ROS in healthy cells that can be stimulated by physical exercise.

Therefore, we suggest focusing on a different approach to specifically target GSCs irrespective of their mutational status and irrespective of their energy metabolism, as has been proposed by Hira et al. [25,26]. GSCs are kept in their hypoxic peri-arteriolar niches in glioblastoma tumors and the SVZ [16] by stromal-derived factor-1α (SDF-1α)–C-X-C receptor type 4 (CXCR4) interactions in a similar way as HSCs and LSCs are kept in their hypoxic peri-arteriolar niches in bone marrow [25,26]. Inhibition of CXCR4 by the FDA-approved drug plerixafor is used successfully to remove LSCs out of the bone marrow niches to render them more sensitive to chemotherapy and HSCs in healthy donors to be harvested in the peripheral blood for stem cell transplantation [26]. Removal of GSCs from their niches in glioblastoma before radiotherapy or chemotherapy may become similarly successful in this context.

## Figures and Tables

**Table 1:** Proteins that are involved in oxidative phosphorylation (OXPHOS) in glioblastoma stem cells (GSCs) as potential selective therapeutic targets.

Protein	Function	Reference
Translocator protein (TSPO)	Mitochondrial transmembrane cholesterol transporter	[66]
Insulin-like growth factor 2 mRNA-binding protein 2 (IGF2BP2)	Delivering of mRNAs to mitochondria	[68]
Glycerol-3-phosphate dehydrogenase I (GPDI)	Prevention of osmotic stress	[69]
Oncostatin M	Cytokine in immunosurveillance	[71]

**Table 2:** Inhibitors of oxidative phosphorylation (OXPHOS) to selectively target glioblastoma stem cells (GSCs).

Inhibitor	FDA-Approved?	Indication	Reference
Atovaquone	Approved	*Pneumocystis jiroveci* pneumonia treatment, malaria prophylaxis	[75]
Mito-Lonidamine (Mito-LND)	Not approved	N/A	[41]
Metformin	Approved	Type 2 diabetes mellitus	[61]
Phenformin	Not approved	N/A	[61]
Verteporfin	Approved	Macular degeneration	[76]

## Data Availability

The data presented in this study are available on request from the corresponding author.
